# Advances in Micro‐Transfer Printing of Lithium Niobate Thin‐Films for Silicon Photonic Devices

**DOI:** 10.1002/nap2.70042

**Published:** 2026-03-20

**Authors:** Margot Niels, Tom Vandekerckhove, Lisa De Jaeger, Maximilien Billet, Bart Kuyken

**Affiliations:** ^1^ Department of Information Technology (INTEC) Photonics Research Group Ghent University—imec Ghent Belgium

**Keywords:** heterogeneous integration, lithium niobate, micro‐transfer printing, silicon photonics

## Abstract

Silicon and silicon nitride waveguide platforms have established themselves as ideal candidates for high‐volume photonic integration, owing to their compatibility with mature semiconductor manufacturing processes. In parallel, lithium niobate has garnered significant attention for its superior electro‐optic and nonlinear properties, positioning it as a key enabler for high‐speed and advanced photonic functionalities. Nonetheless, the heterogeneous integration of lithium niobate with silicon photonics remains a technical challenge and an active area of investigation. We provide a comparative analysis of integration methods, detail the microtransfer printing process flow and highlight key demonstrators, including modulators, nonlinear devices and emerging applications such as optomechanics and quantum photonics. The scalability of microtransfer printing is discussed in the context of high‐volume manufacturing. This review underscores the potential of microtransfer printing to bridge the gap between high‐performance lithium niobate devices and industrial‐scale photonic integration.

## Graphical abstract

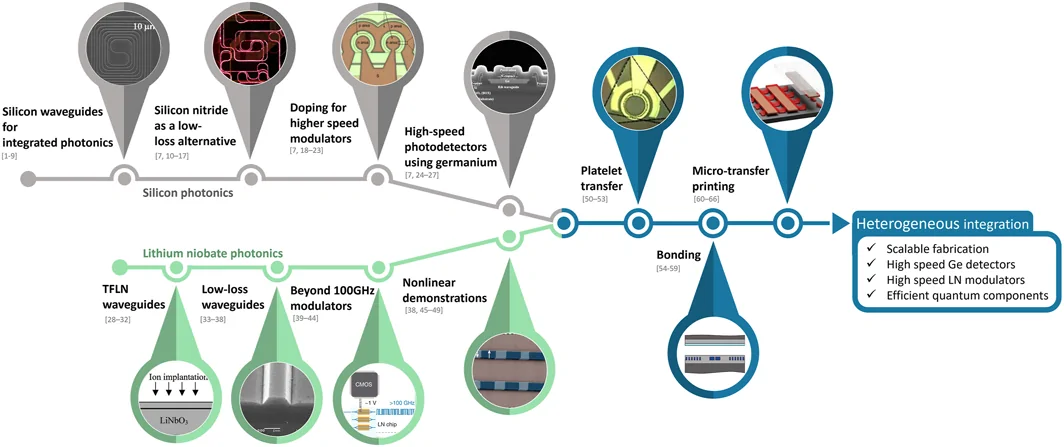

## 1 Introduction

Integrated photonics has rapidly evolved into a cornerstone technology for applications ranging from telecommunications and quantum optics to biosensing and RF systems [1]. This evolution has been driven by the convergence of photonic functionality with semiconductor manufacturing, enabling the development of photonic integrated circuits (PICs) that mirror the scalability and compactness of their electronic counterparts.

Early efforts focused on CMOS‐compatible waveguides, enabling high‐volume fabrication as they could leverage the existing manufacturing processes and know‐how of silicon electronics and the CMOS industry [2, 3]. Thereto, silicon photonics (SiPho) emerged as a leading platform [4, 5, 6, 7, 8, 9, 10, 11], offering dense integration, transparency in the commercially important infrared wavelength bands and high‐speed components such as ring modulators and photodetectors. Silicon nitride (SiN) waveguides soon followed, valued for its ultralow loss and even broader transparency window, allowing it to operate in lower wavelengths, enabling applications in life sciences [12, 13, 14, 15, 16, 17]. Since SiN has virtually no two‐photon absorption, it is more suitable for nonlinear processes than Si [2]. One could imagine that the combination of the two platforms brings together the best of both worlds [2, 13, 18], which is already commercially available on 300‐mm wafers. In addition to the basic passive components of the platform, such as waveguides, splitters, modulators, ring resonators and heaters [19, 20, 21, 22, 23, 24], more intricate building blocks emerged, delivering optical filters and high‐speed photodetection (germanium (Ge) photodetectors) to the platform [25, 26, 27, 28]. However, other materials remain much more efficient in providing gain and both Si and SiN have a negligible Pockels effect and second‐order nonlinearities, which makes fast modulation and second harmonic generation much harder.

Recently, with lithium niobate on insulator (LNOI) becoming commercially available, LN has come more into the picture for many researchers [29, 30, 31, 32, 33, 34, 35, 36, 37, 38] as an alternative platform. Even though its need for specialised foundries, integrated photonics on thin‐film lithium niobate (TFLN) is an emerging and rapidly advancing platform [39]. As the only platform that simultaneously supports low propagation loss [29, 40, 41], fast optical modulation at low‐voltage operation [42, 43, 44, 45, 46, 47] and efficient all‐optical nonlinearities [41, 48, 49, 50, 51, 52], it poses as a great candidate for telecom, datacom and quantum technologies [53]. With fabs offering commercial devices and multiproject wafers on 150 mm wafers [54, 55, 56, 57], one could say also the industrialisation of the TFLN platform is on a good way. However, scalable fabrication is not as straightforward as with more CMOS‐compatible platforms (SiPho) [58], making LN a more expensive technology [53, 59].

Rather than choosing one over another platform, there is a trend towards combining several platforms, as illustrated in Figure 1. In that way, the ultralow‐loss SiN can be combined with the dense Si integration, the gain from a III–V material and high‐speed or nonlinear LN [78, 79, 80, 81]. In general, there are two main groups of cointegration techniques: hybrid integration, where two PICs of different platforms are integrated next to [82, 83, 84, 85] or on top of each other [86, 87] after full fabrication; and heterogeneous integration, where several material technologies are combined onto a single PIC [88]. Since the PICs are first produced separately, hybrid integration techniques (i.e., flip‐chip bonding, photonic wire bonding and butt coupling) offer the known‐good‐die principle where characterised PICs of good quality can be cherry picked before integration. The separate PICs can also offer some flexibility where you combine whatever functionalities you need with different building blocks. One can imagine that, just such as in the electronics industry, this method is highly accessible automated production [82], albeit with limited throughput since active alignment and integration have to be done PIC by PIC. On top of that, these assemblies are much larger compared to heterogeneously integrated PICs. The smaller size and smaller costs due to the economy of scale make heterogeneous integration techniques (i.e., epitaxial growth, 2D‐transfer, die‐to‐wafer or wafer‐to‐wafer bonding, microtransfer printing and deposition) a better candidate for high‐volume applications [89]. Also higher alignment accuracy, closer integration (ergo lower losses when transitioning between the different materials) and similar performance and functionalities as monolithic platforms make this group of techniques more attractive. However, the disadvantage is the need for very smooth and clean surfaces when making these heterogeneous platforms [88].

**FIGURE 1:**
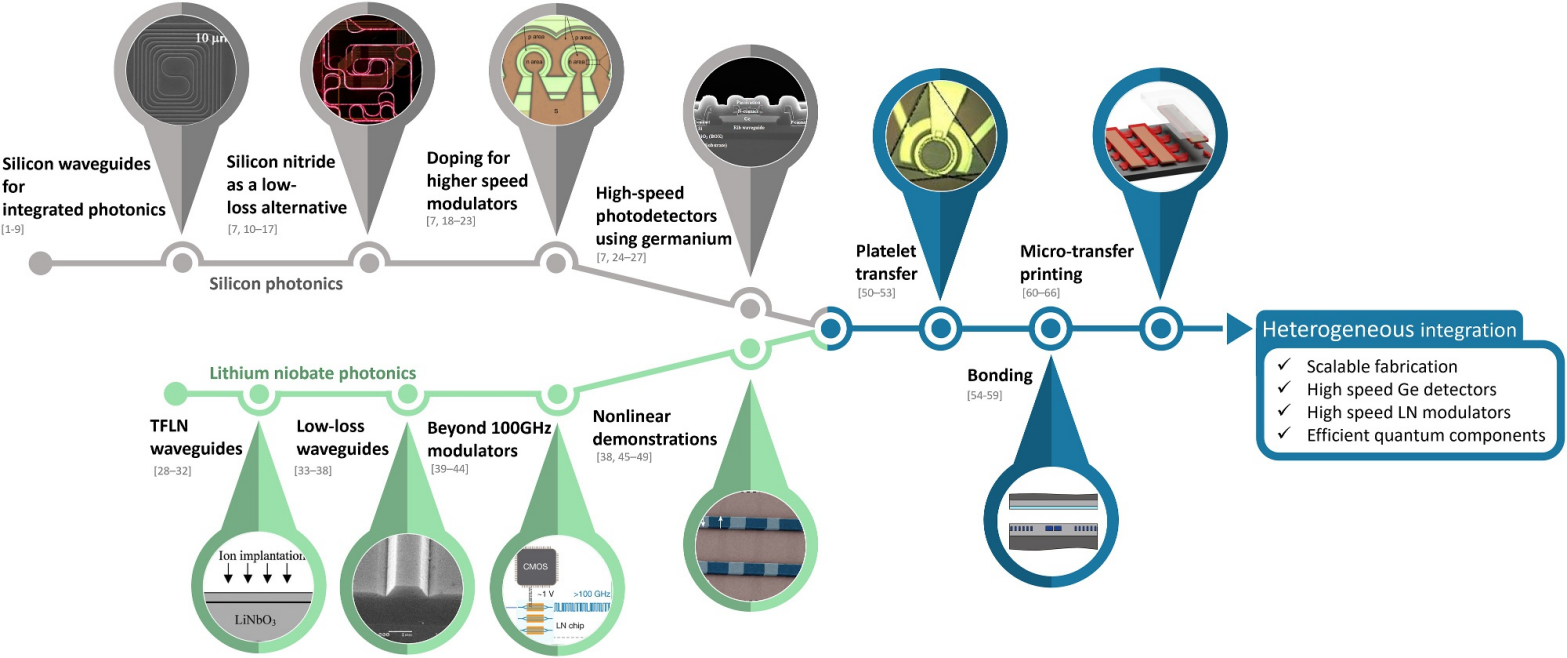
Two high‐performance platforms blend into one through heterogeneous integration. Silicon photonics, with low‐footprint Si waveguides [3, 4, 5, 6, 7, 8, 9, 10, 11], low‐loss SiN waveguides [2, 4, 12, 13, 14, 15, 16, 17, 18] and high‐speed modulators [4, 19, 20, 21, 22, 23, 24], and high‐speed photodetectors [4, 25, 26, 27, 28], is merged with lithium niobate photonics [31, 35, 36, 37, 38], known for its low‐loss waveguides [29, 34, 39, 40, 41, 60], ultrahigh‐speed modulators [42, 43, 44, 45, 46, 47] and efficient nonlinear components [41, 48, 49, 50, 51, 52], through platelet transfer [61, 62, 63, 64], die‐to‐wafer or wafer‐to‐wafer bonding [65, 66, 67, 68, 69, 70] or microtransfer printing [71, 72, 73, 74, 75, 76, 77] to create a platform which still benefits largely from the CMOS compatibility, enables high‐speed modulation and photodetection and houses efficient quantum components. The figure on silicon nitride is adapted with permission from ref. [13]; the image on doping for higher speed modulators is adapted with permission from ref. [22] Optical Society of America; the depiction of high‐speed Ge photodetectors is adapted with permission from ref. [26] Optical Society of America; the picture on TFLN waveguides is reproduced from ref. [35], with the permission of AIP Publishing; the SEM image of low‐loss LN waveguides is adapted with permission from ref. [39] Optical Society of America; the schematic on fast modulators is adapted from ref. [42] Nature; the photograph on nonlinear demonstrations is adapted with permission from ref. [48] Optical Society of America; the one on platelet transfer is adapted with permission from ref. [64] Optical Society of America; the picture on bonding is adapted from ref. [66] under licence CC BY 4.0 and the figure on μTP is adapted with permission from ref. [77] Optica Publishing Group.

This review focuses on one of the more recent heterogeneous integration techniques for incorporating lithium niobate onto SiPho platforms: microtransfer printing (μTP). The following section provides a brief overview of the principal heterogeneous integration approaches for TFLN on SiPho, with a comparative analysis of μTP. Thereafter, the μTP process flow is examined in detail. Subsequently, published demonstrators are reviewed and categorised into three subsections: modulators, nonlinear devices and other closely related material demonstrations. This is followed by a discussion on the scalability of μTP, outlining its transition from a research‐oriented technique to a viable process for industrial‐scale production. The review concludes with a forward‐looking perspective and final remarks.

## 2 Heterogeneous Integration Techniques for LN on SiPho

This section provides a comparative overview of heterogeneous integration techniques for LN on SiPho. It begins with a brief description of each method, starting with microtransfer printing (μTP), which is the central focus of this review. The techniques, also depicted in Figure 2 along with some demonstrators, are subsequently evaluated against several key factors, including process flexibility, compatibility with existing SiPho platforms, scalability and alignment accuracy.

**FIGURE 2:**
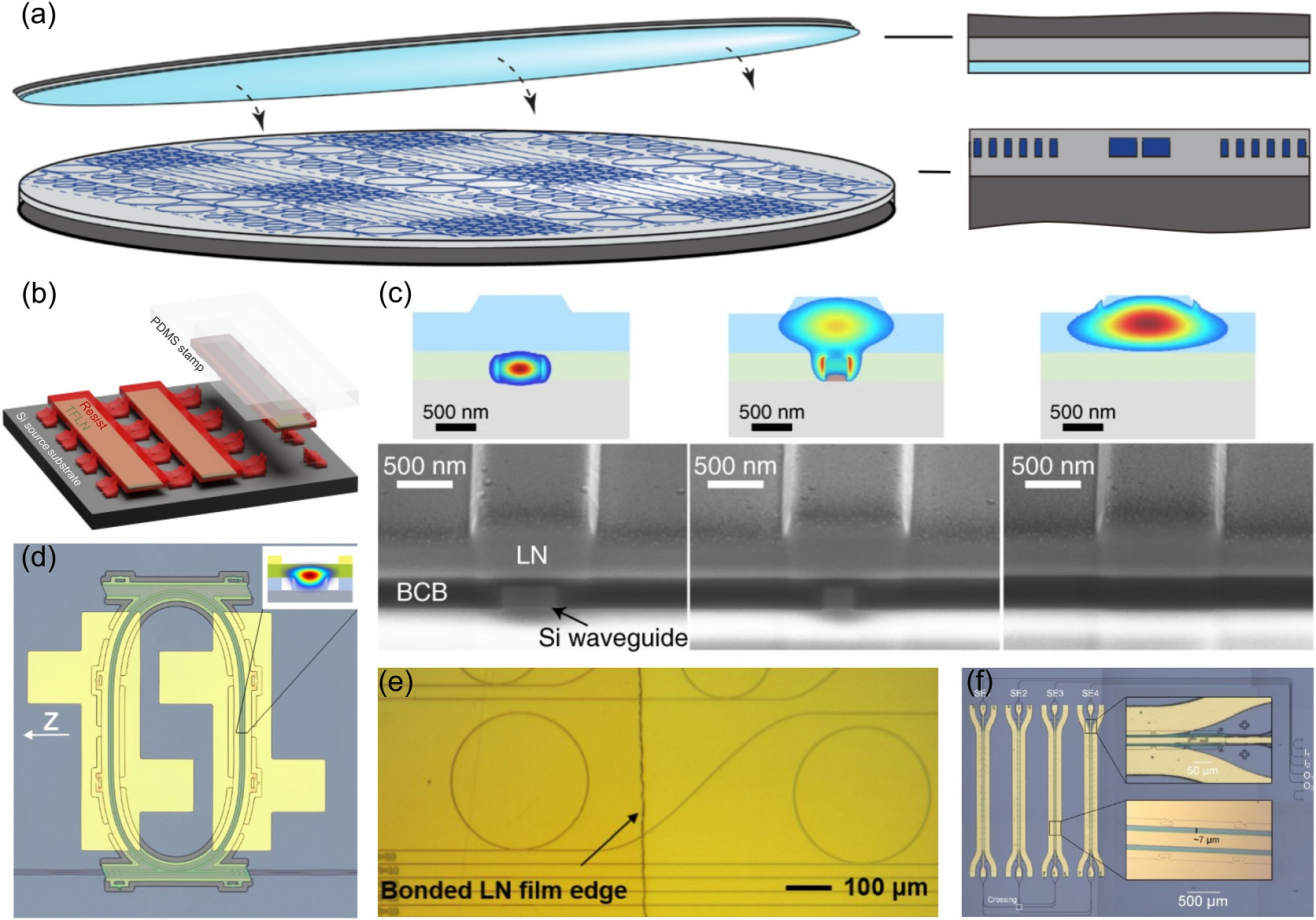
Heterogeneous integration techniques of (a) wafer bonding [66] and (b) microtransfer printing [77]. (c) The heterogeneous mode propagating from the SiPho layer to the integrated LN layer [68]. Demonstrations of (d) a microtransfer printed heterogeneous ring resonator [90], (e) die‐to‐wafer bonded heterogeneous circuitry [91] and (f) a switch circuit with microtransfer printed Mach–Zehnder heterogeneous modulators [92]. Panel (a) is adapted from ref. [66] under licence CC BY 4.0; panel (b) is adapted with permission from ref. [77] Optica Publishing Group; panel (c) is adapted with permission from ref. [68] Nature; panel (d) is adapted with permission from ref. [90] Optica Publishing Group; panel (e) is adapted with permission from ref. [91] Optica Publishing Group and panel (f) is adapted with permission from ref. [92] IEEE.

Microtransfer printing is a pick‐and‐place technique used to integrate suspended microscale devices—commonly referred to as coupons—from a source substrate onto a target substrate [71, 72, 73, 77]. After preparing both source and target substrates separately (more details in the next section), the actual printing process begins by retrieving the coupon using a soft elastomeric stamp, typically made of polydimethylsiloxane (PDMS), which temporarily adheres to the device through van der Waals forces. Then, this stamp, with the coupon attached to it, is transferred to the target site and the coupon is optically aligned to the target circuitry. Once aligned, the coupon is released onto the target substrate, where it bonds via the same intermolecular forces. However, successful transfer relies heavily on surface quality: if the topography of the target substrate or the underside of the coupon exhibits excessive roughness, the van der Waals attraction may be insufficient to overcome the adhesion between the stamp and the coupon. To mitigate this, a buffer layer, benzocyclobutene (BCB), is applied to the target surface prior to printing, which smooths out surface irregularities. Moreover, heating the target substrate during the printing step softens the BCB even more, promoting stronger contact between the coupon and the target. More exotic derivates of μTP exist but were not reported for the integration of LN on SiPh yet [93].

It is worth noting that μTP is a relatively recent development and several other techniques for LN‐SiPho cointegration have been proposed in literature prior to its emergence. The first approach, colloquially known as platelet transfer, involves the pick‐and‐place of exfoliated LN thin films using a fibre tip [61, 62, 63]. This approach employs ion‐slicing, also known as Smart Cut and used to create LNOI [31, 94, 95], in combination with thermal treatments to create TFLN microplatelets from bulk single‐crystal LN. These thin films, which are strip‐ and triangular‐shaped and are varying in size, are then bonded directly to the SiPho target using a fibre tip on a probe station. Sometimes BCB is used as an intermediate layer [63] for smoothening the surface, just such as in the μTP method. The final step involves annealing the heterogeneous structure to complete the bonding process and cure the BCB layer (typically at 280°C), if present. The transferred flakes are unpatterned, relaxing the alignment constraints.

The most widely adopted approach remains wafer‐to‐wafer or die‐to‐wafer bonding [65, 66, 67, 68, 69, 70, 91]. In this technique, a die or a wafer of LNOI is directly bonded face‐down onto the SiPho target substrate. BCB can also be used as an adhesive or buffering layer since, just such as for μTP, the bonding needs a smooth surface. Following bonding, the structure is annealed and the substrate and BOX are removed. The exposed LN layer can then be processed to fabricate photonic devices directly atop the SiPho platform.

Compared to wafer bonding, platelet transfer and μTP stand out as versatile and modular approaches for heterogeneous photonic integration, as it enables the co‐placement of devices with varying crystalline orientations and layer thicknesses within a single layout, offering design flexibility. On top of that, they both support the known‐good die principle, allowing defective coupons to be identified and excluded prior to integration, thereby maximising device yield. In more intricate multi‐layer SiPho platforms, recesses can be used to accommodate LN material via μTP or platelet transfer to interact with deeper‐laying layers. The low‐temperature postprocessing of both approaches reduces the thermal stress and broadens compatibility with temperature‐sensitive materials. Moreover, these pick‐and‐place methods allow LN to be only deposited where its functionality is required, optimising material usage and reducing source wafer consumption. The techniques are, furthermore, compatible with any wafer size, support multimaterial cointegration and relax full‐wafer flatness constraints since local planarisation is sufficient for successful bonding. It should be noted that the feasibility of multimaterial cointegration in μ TP is still under investigation, although the complexity of coordinating the different process flows of each material is already shown when printing III/V on SiN platform, where also the Si interposer is printed prior to the III/V device [96, 97].

Microtransfer printing, in particular, benefits from decoupled process flows between source and target wafers, allowing prefabricated devices to be integrated after CMOS back‐end processing whilst preserving the integrity of the target wafer, thus ensuring full CMOS compatibility. This also applies to platelet transfer as long as no circuits are needed. In case routing is needed in the transferred TFLN, postbonding processing is necessary, just such as in the wafer bonding approach. Nevertheless, a notable advantage of the platelet transfer technique is that LN thin films can be derived directly from bulk single‐crystal LN, thereby eliminating the need for LNOI substrates. However, the uncontrolled size of the microplatelets makes it impossible to scale this technique to mass‐production volumes.

Die‐to‐wafer and wafer‐to‐wafer bonding remain the most mature and widely adopted techniques for LN on SiPho integration, offering scalability and process uniformity. These techniques enable the transfer of entire LN surfaces in a single bonding step, eliminating the need for high‐precision alignment between source and target wafers and allowing for parallel integration. This not only simplifies the integration process but also significantly reduces per‐device cost by supporting batch fabrication and minimising handling complexity. Up to now, the availability of LNOI wafers was limited to 150 mm diameters, constraining their use in larger‐scale SiPho platforms. However, the recent commercialisation of 200 mm LNOI wafers has begun to address this limitation, paving the way for broader adoption in next‐generation photonic foundries. A key advantage of wafer bonding lies in its support for post‐bonding LN processing, which allows for high‐resolution patterning and device definition directly atop the silicon photonic circuitry. This facilitates compact, high‐performance integration of modulators, filters, and nonlinear components with minimal optical loss and excellent alignment fidelity. However, the extra processing of the LN on top of the SiPho layers cannot be done in the same machinery as the CMOS wafer due to Li contamination concerns. This involves high‐resolution deep UV steppers and dedicated etching tools as this is a front‐end‐of‐line process. However, transfer‐printing allows for back‐end‐of‐line process which does not involve structures with small critical dimensions. Therefore, less dedicated tools are needed.

Despite its versatility, μTP still faces several practical challenges that currently limit its widespread industrial deployment. The technique is exceptionally attractive for research and development (R&D) environments, where layout reconfigurability, material efficiency, and selective integration are essential. However, scaling μTP beyond its R&D origins into high‐volume manufacturing remains an active area of development.

A first bottleneck is throughput. μTP was historically viewed as a sequential pick‐and‐place process, which raised concerns about scalability compared to wafer‐bonding or die‐to‐wafer transfer. However, recent demonstrations of multicoupon transfer (array‐printing) have shown that parallelisation is feasible and can substantially increase throughput [74]. Similarly, the successful transfer of centimetre‐scale LN coupons onto Si photonic platforms [77, 98] indicates that μTP is not inherently limited to small device footprints.

A second challenge is alignment precision. μTP requires accurate placement of coupons relative to underlying photonic structures, which can be more demanding than platelet transfer or wafer bonding. Nevertheless, state‐of‐the‐art μTP tools now report placement accuracies on the order of 500 nm (3σ) [99], which is sufficient for high‐performance photonic integration when adiabatic interlayer couplers are properly designed.

A third bottleneck relates to ecosystem maturity and supply‐chain readiness. μTP is a relatively new heterogeneous integration technology, and only a limited number of commercial foundries currently support it. This restricts access to volume manufacturing and increases the burden on research groups to develop in‐house processes. However, early industrial adoption, such as the offering of μTP services by X‐Fab, demonstrates that commercial uptake is progressing and that the technique is compatible with established semiconductor workflows.

An overview of the comparison of these techniques is collected in Table 1. Overall, although μTP introduces unique integration challenges, ongoing advances in tool accuracy, array‐printing, large‐area coupon transfer and foundry adoption indicate that these bottlenecks are being progressively mitigated.

**TABLE 1:** Heterogeneous integration techniques versus their key properties.

Technique versus key‐property	Source material usage	Thermal budget	Wafer‐scale integration	Reaching deeper SiPho layers	Process uniformity	Design flexibility	Needed alignment accuracy	Known‐good die	Proto‐typing
Microtransfer printing	Low	Low	Under R&D	Straight‐forward	Moderate‐high	High	High	Possible	High
Die/wafer‐to‐wafer bonding	High	High	Shown	Hard	High	Low	Low	Not possible	Low
Platelet transfer	Low	Low	Limited	Straight‐forward	Low	High	Moderate	Possible	Moderate

## 3 Fabrication Methods for μTP

The general process flow of μTP has been introduced in the previous section, outlining its role as a versatile pick‐and‐place technique for integrating LN onto SiPho platforms. The following sections explore the practical implementation of μTP in greater detail, beginning with substrate preparation, followed by the printing procedure itself. Particular attention will be given to the fabrication strategies for suspended LN coupons, the tethering mechanisms that enable their release and the post‐transfer processing required to render the devices functional.

### 3.1 Source and Target Wafer Preparation

Before μTP can take place, both the source and target substrates must be carefully fabricated. The target wafer generally imposes minimal design constraints. This allows it to be prepared using standard CMOS‐compatible techniques, which will not be discussed in detail in this review. In some reports, an oxygen plasma treatment is applied prior to printing on the target substrate to activate the surface or for meticulous cleaning [76, 92, 100, 101, 102]. As previously mentioned, a buffer layer may also be deposited before printing to enhance the bonding strength by further reducing surface roughness.

In contrast, the source wafer requires more intricate processing as the devices (or coupons) must be suspended to enable successful pick‐up and transfer. These suspended structures remain temporarily anchored to the source chip (or wafer) via tethers, which are designed to break upon contact with the PDMS stamp, thereby releasing the coupon for transfer. For LN, more specifically, there are a few methods described in the literature to transform the LNOI wafers into suspended coupons, differing primarily in the tether material and suspension strategy. Figure 3 provides an overview of these techniques, which will be clarified sequentially, starting from the top row and proceeding downward.

**FIGURE 3:**
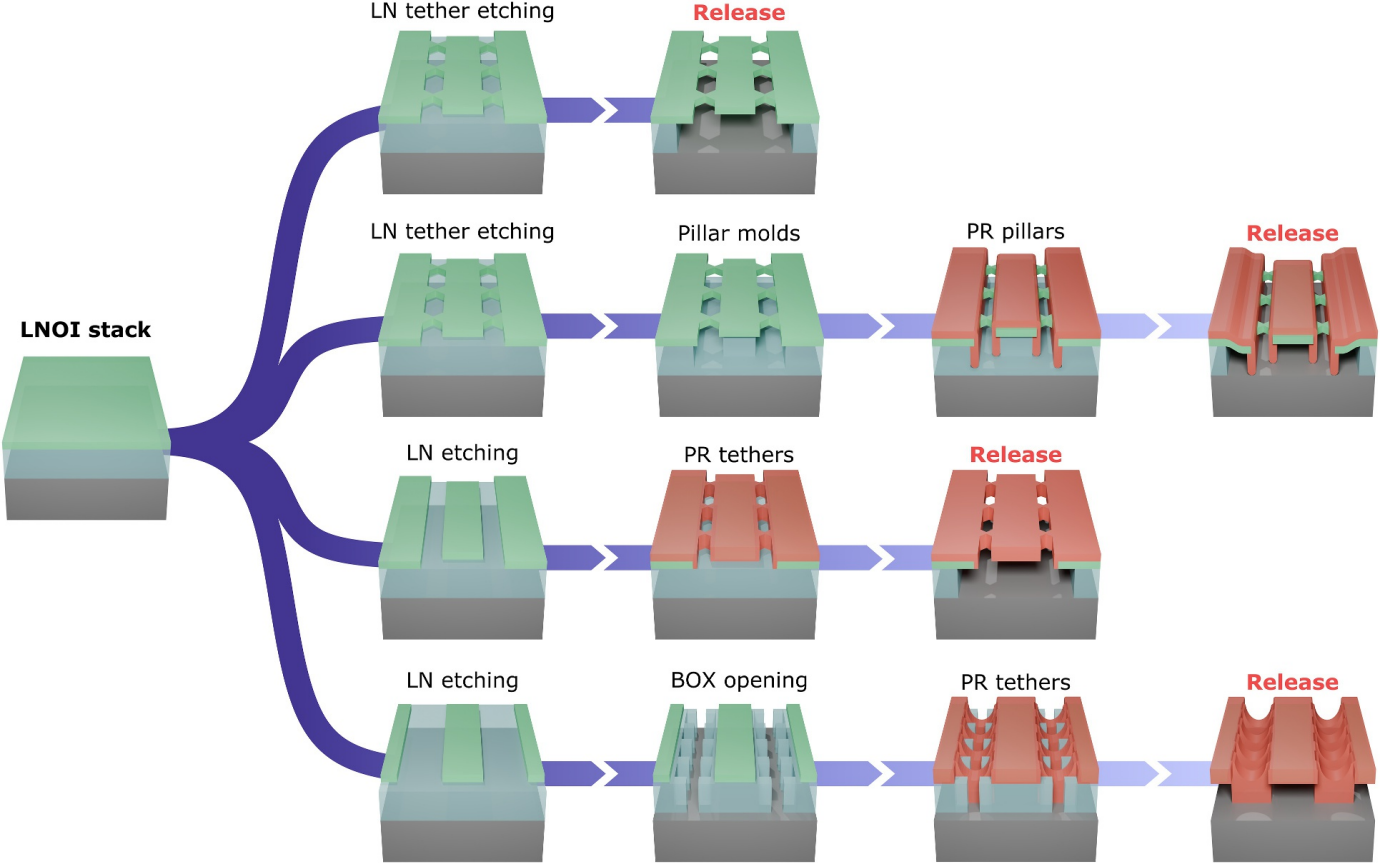
Schematic overview of the different approaches of coupon fabrication on the source sample, starting from an LNOI sample. Top to bottom: LN tethers to the side, LN tethers to the side with support structures (pillars), photoresist resist (PR) tethers to the side and PR tethers to the substrate.

The approaches of Vandekerckhove et al., De Jaegere et al., Vanackere et al., Palici et al., Murai et al. and Su et al. [73, 75, 90, 92, 99, 100, 102, 103] rely on monolithic LN tethering, utilising the LN itself to make the tethering structures. When etching the coupons in their final device shape, they are not completely separated from the surrounding LN but are still attached through fragile LN bridges (tethers) to the side. In the approach of Su et al., Palici et al. and Murai et al. [92, 100, 102, 103], the membranes are then undercut by etching the buried oxide (BOX) away using an HF‐based wet etchant. However, Vandekerckhove et al. [73] showed that during the liquid‐to‐vapour transition after this HF etching, capillary forces could cause the coupons to collapse to the substrate. To mitigate this, they introduced support structures that maintain suspension during drying. These supports are created by partially etching into the BOX layer (also using HF), followed by the application of photoresist, which forms pillars beneath the coupon. These pillars can be placed at the edges of the coupon or within etched holes in the device itself, allowing for internal support, an approach particularly effective for wider coupons. To accommodate these bottom‐facing pillars during transfer, trenches must be etched into the target wafer, providing mechanical clearance and positional guidance. Although this adds an extra processing step to the target substrate, it also enhances alignment accuracy as the pillars must fit precisely into the trenches before the coupon can be printed.

Beyond LN‐based tethering, resist‐based tethering methods have also been explored. In these approaches, the LN device is first fully etched. Li et al. [72] then coat the entire source wafer with photoresist and develop trenches around the coupon, leaving narrow resist bridges that connect the coupon to the surrounding material, functionally similar to LN tethers but composed of polymer. As in Su et al.’s method, the BOX is subsequently removed using an HF‐based etchant, releasing the coupon. An alternative resist tethering strategy involves anchoring the coupon to the substrate rather than the sides as described by Niels et al., Badri et al., Tan et al., Anathachar et al. and Sumon et al. [74, 76, 77, 101, 104, 105]. In this method, the BOX is first etched down to the substrate before applying the resist, after which the resist is patterned to cover the entire coupon whilst forming tether structures that extend downward, anchoring the device to the substrate surface. Afterward, the BOX is etched away with an HF‐based wet etchant and the coupons are ready to be picked. Although resist tethering may appear more cumbersome and less elegant than LN tethering, it also offers distinct advantages. Due to concerns over lithium contamination and that the brittle LN tethers are prone to premature breakage, polymer‐based tethers can be seen as a safer and more controllable alternative in certain fabrication environments. Due to high aspect ratios, some might be concerned about warping or mechanical stress, but none of these issues have been reported in the literature for thin layers of LN. Also, the surface roughness of the produced coupons is sometimes questioned but was reported by Vandekerckhove et al. to be in the same range as the LNOI source wafer before fabrication [75].

### 3.2 Printing Procedure

During the actual printing procedure, both the source and the target substrates are mounted on chucks in the μTP machine, whereas a PDMS stamp is affixed to a glass plate on the transfer head. This PDMS stamp is mostly rectangularly shaped; however, Su et al. [100] demonstrate that it could be custom‐shaped to match the geometry of the device—in their case a ring—allowing for a more targeted transfer. Through a camera above the glass plate on the transfer head, the PDMS is aligned with the desired coupon on the source substrate and slightly pushed against it to establish adhesion before picking it up, as illustrated in Figure 4a. The coupon is thereafter transferred to the target side, where it is carefully aligned to the target structure and pressed onto the surface to initiate bonding, after which the stamp is retracted (Figure 4b). This placement step can be done at elevated temperature to enhance the bonding strength and surface conformity, but is not reported in any studies on μTP of LN on SiPho platforms. Although not all papers explicitly state that the printing happened at room temperature, Su et al. and Badri et al. [76, 92, 100] confirm that successful transfer is achievable at room temperature.

**FIGURE 4:**
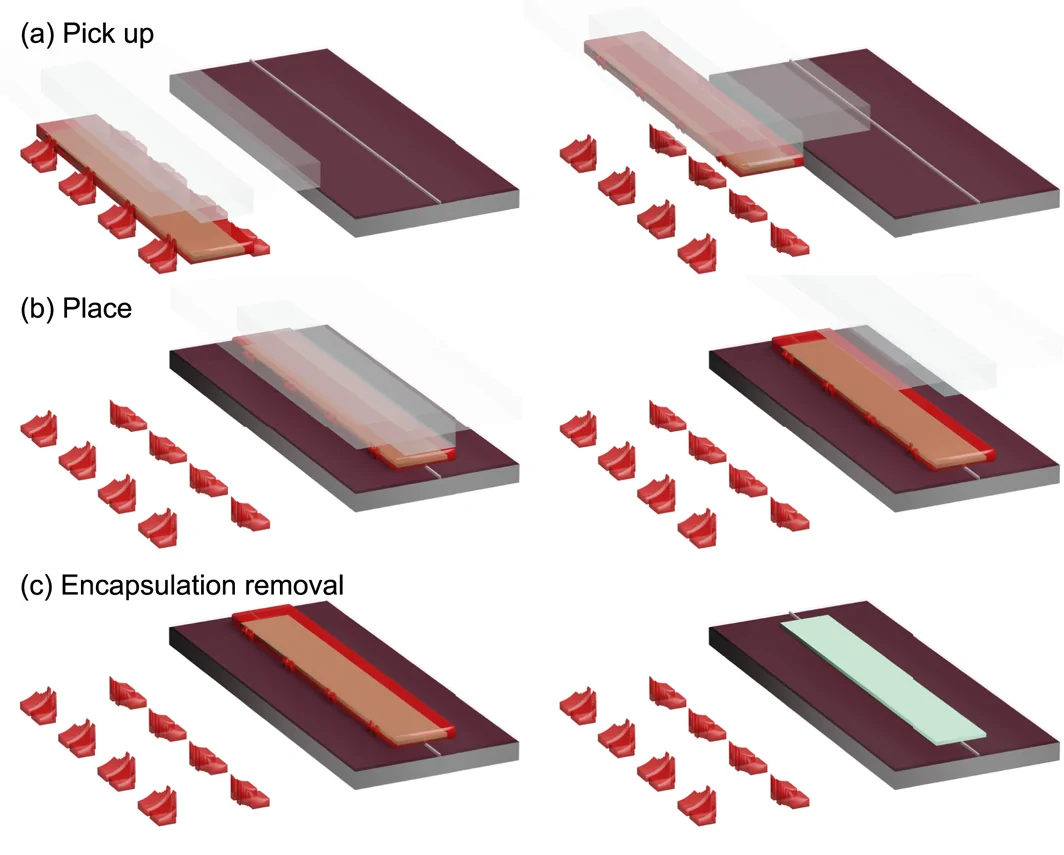
Process flow for the integration of LN using transfer printing. (a) The coupon is picked up from the source sample, (b) then transferred to the target sample and after printing and if needed, (c) the resist encapsulation is removed.

Following the printing step, postprocessing is required to render the device functional. First, as depicted in Figure 4c, if photoresist encapsulation was used, the encapsulation needs to be removed. This can be achieved via a wet etch using acetone [76], a dry etch using oxygen plasma [72, 74, 77, 101] or a combination of both [75, 104]. Subsequent processing steps depend on the intended application of the printed device. For example, if redistribution layers (RDL) are needed, metallisation is applied to the heterogeneous structure [73, 74, 76, 77, 90, 92, 99, 100, 102, 104]; there could be a curing step, which may [74, 75] or may not [72] be for enhancing transparency of the buffer layer or some wet etching and oxide cladding can be applied for tuning [75]. A more detailed overview of the postprinting processing steps will be provided in the following sections, where all reported applications of μTP of LN on SiPho platforms are discussed.

In Table 2, the details of the used tethering method, target stack details, coupon and sizes and the number of printed coupons are listed for all known demonstrations of μTP of LN on SiPho. Note that the usage of an adhesive is not always specifically mentioned in the article and the number of coupons is deduced from the number of coupons visible in pictures if the exact number was not mentioned (N.M.) in the text.

**TABLE 2:** Overview of all printed LN coupons for heterogeneous LN/SiPho platforms.

Ref	Tether method	Platform	Coupon size (L × W × H)	Adhesive	Target size	# Coupons	Printing temperature
[71]	LN to the side	LN/SiN	1 mm × 60 μm × 300 nm	BCB	Chip	1	N.M.
[72]	Resist to the side	LN/SiN	0.04 mm × 40 μm × 600 nm	None	Chip	1	N.M.
[73]	LN tethers + pillars	LN/SiN	1 mm × 40 μm × 300 nm 1 mm × 120 μm × 300 nm	N.M.	Chip	25	N.M.
[74]	Resist to the substrate	LN/SiN + Si	7 mm × 30 μm × 300 nm	BCB	Wafer	217 + 1^a^	N.M.
[75]	LN tethers + pillars	LN/SiN	1 mm × 40 μm × 300 nm	PBCB	Chip	5	N.M.
[76]	Resist to the substrate	LN/SiN	2.4 mm × 100 μm × 300 nm	None	Chip	3	Room temperature
[77]	Resist to the substrate	LN/SiN	10 mm × 30 μm × 300 nm	None	Chip	25 + 1^a^	N.M.
[90]	LN tethers + pillars	LN/SiN	0.5 mm × 250 μm × 300 nm	N.M.	Chip	1	N.M.
[92]	LN to the side	LN/SiN	2 mm × 50 μm × 300 nm	N.M.	Chip	6	Room temperature
[99]	LN tethers + pillars	LN/SiN	2 mm × 30 μm × 300 nm	N.M.	Chip	27 + 5^a^	N.M.
[100]	LN to the side	LN/SiN	0.75 mm × 250 μm × 300 nm 2 mm × 230 μm × 300 nm		Chip	15 + 52^a^	Room temperature
[101]	Resist to the substrate	LN/SiN	1.2 mm × 70 μm × 200 nm	N.M.	Chip	5	N.M.
[102]	LN tethers	LN/Si	3 mm × 100 μm × 200 nm	None	Chip	1	N.M.
[103]	LN tethers	LN/SiN	0.11 mm × 60 μm × 170 nm	N.M.	Chip	1	N.M.
[104]	Resist to the substrate	LN/Si	0.7 mm × 50 μm × 600 nm	N.M.	Chip	6	N.M.
[105]	Resist to the substrate	LN/SiN	2.4 mm × 100 μm × 300 nm	N.M.	Chip	2	N.M.

Abbreviations: BCB, benzocyclobutene; PBCB, photosensitive benzocyclobutene.

^a^ Failed or dummy coupons.

## 4 Demonstrators Using μTP of LN on SiPho

An increasing number of demonstrators have been reported in the literature, showcasing the application of μTP for the heterogeneous integration of LN onto SiPho platforms. Since all demonstrators use X‐cut LN, the X‐cut will be omitted for the rest of the text. This section provides a structured overview of all published demonstrators, grouped by device category. The first subsection delves into μTP‐enabled modulators, which form the most extensively studied class of demonstrators in the literature. Their prominence stems from the relative maturity of modulator design and the compatibility of μTP with key modulator architectures. Within this category, three subtypes will be examined: Mach–Zehnder modulators (MZMs), the most widely reported, owing to their straightforward implementation and robust performance in μTP‐based integration; Ring modulators, which offer compact footprints and energy‐efficient operation, and have emerged as a popular alternative in recent studies and Fabri–Pérot (FP) modulators, a less explored category, currently limited to a single proof‐of‐concept publication, yet noteworthy for their unique resonance‐based modulation mechanism.

The second subsection highlights second‐harmonic generation (SHG) devices, reflecting the growing interest in nonlinear photonic applications. Although fewer in number of publications compared to modulators, SHG demonstrators underscore the potential of μTP to unlock advanced capabilities beyond conventional linear photonics. To conclude, emerging directions that extend beyond the core scope of this review will be briefly explored. Comparisons will be drawn with demonstrators that fall slightly outside the conventional LN‐on‐SiPho framework, illustrating the broader versatility and adaptability of μTP.

### 4.1 Modulator Devices

#### 4.1.1 Mach–Zehnder Modulators

Mach–Zehnder modulators (MZMs) have become the de facto demonstrators for heterogeneously integrated LN on SiPho circuits, owing to their high bandwidth, well‐understood architecture, straightforward characterisation and direct sensitivity to electro‐optic phase shifts. Their interferometric nature allows clear benchmarking of material performance, whereas their compatibility with high‐speed modulation and scalable electrode designs makes them ideal for evaluating integration processes such as μTP. Within the context of μTP, two principal configurations have been employed: single‐ended and differential MZMs.

The first‐ever demonstrator of μTP for LN on SiPho utilised a single‐ended MZM in a ground‐signal (GS) configuration on one arm of the interferometer [71]. Vanackere et al. established the feasibility of LN printing by suspending LN coupons via lateral tethers (see Figure 5a). A 1 mm × 60 μm × 300 nm LN coupon was printed onto a SiN target, covered with BCB. Electrodes were subsequently deposited atop the heterogeneous SiN/LN stack, with a length of 0.9 mm, thereby limiting the interaction length between the optical mode and the electric field. Passive and active characterisation in the C‐band revealed an extinction ratio (ER) of 12 dB, a half‐wave voltage (Vπ) of 61.1 V (with a 6.5 μm electrode gap) and an insertion loss (IL) of 7 dB, inclusive of SiN routing. Although bandwidth measurements were not performed, the linear phase response, maintained even under polarity reversal of the bias voltage, confirmed modulation via the electro‐optic effect.

**FIGURE 5:**
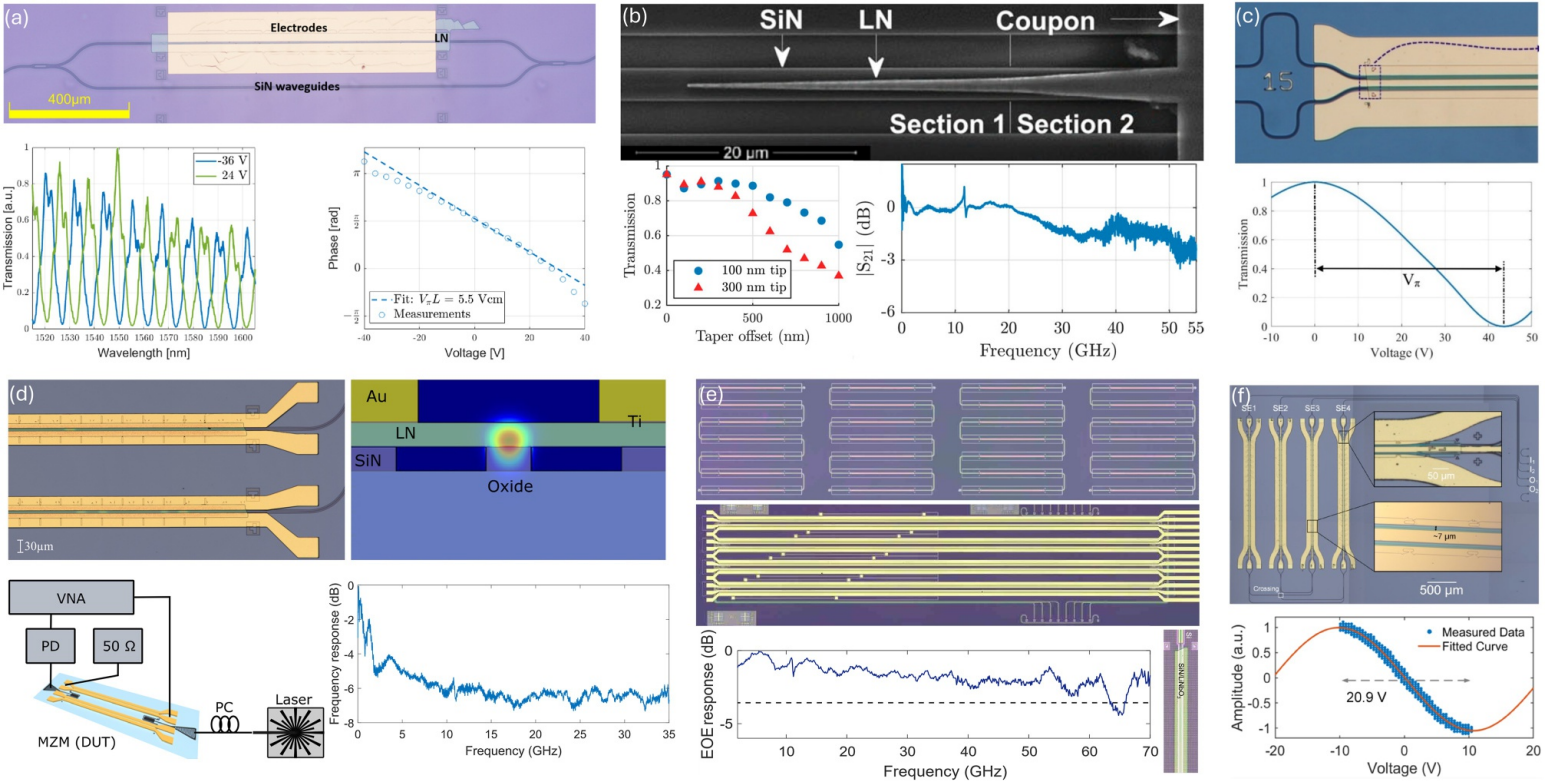
Reported microtransfer printed LN on SiPho Mach–Zehnder modulators. (a) First demonstrated device and its Vπ [71]; (b) refined design, including tapers in the LN, to minimise insertion loss and its high‐speed behaviour [99]; (c) more compact design and its Vπ [76]; (d) centimetre‐long coupons with high length‐width ratio with a crossection of the simulated mode, the electric‐optic‐electrical measurement setup and high‐speed data measured with that setup [77]; (e) a demonstration on mass‐printing with an array of coupons printed in one cycle, four MZMs, a high‐speed response of one of them and a close‐up of a transfer printed coupon [74]; (f) an optical switch using MZMs and the measured Vπ of one such an MZM [92] . Panel (a) is adapted with permission from ref. [71] IEEE; panel (b) is adapted from ref. [99], licenced under a Creative Commons Attribution (CC BY 4.0) licence; panel (c) is adapted from ref. [76] with licence CC BY 4.0; panel (d) is adapted with permission from ref. [77] Optica Publishing Group; panel (e) is adapted with permission from ref. [74] Optica Publishing Group and panel (f) is adapted with permission from ref. [92] IEEE.

The design of the modulator and the coupons were refined in the following years, which led to a C‐band differential GSSG configuration with optimised electrode design and tapered LN coupons (shown in Figure 5b), which were suspended using the pillar method [73, 99]. This approach improved yield and reduced transition losses (i.e., losses incurred at the SiPho–LN interface), achieving 3.3 dB IL including SiN routing. LN coupons were now printed on both arms, with opposing orientations, and their dimensions were adjusted to 2 mm × 30 μm × 300 nm, doubling the interaction length and halving the width to facilitate suspension. The electrode gap was reduced to 6 μm, and a thinner SiN waveguide was employed to enhance modal overlap with the LN. These optimisations yielded a Vπ of 14.8 V and an ER of 39 dB. High‐speed characterisation using a vector network analyser (VNA) and photodiode revealed a 3 dB electro‐optic–electrical (EOE) bandwidth^1^ of at least 55 GHz, limited only by the noise floor due to the high Vπ (trace depicted in Figure 5b). Furthermore, 27 out of 32 coupons were printed with ≤500 nm 3σ misalignment, indicating promising yield metrics.

A subsequent demonstration targeted low driving voltage by printing centimetre‐scale LN coupons (10 mm × 30 μm × 300 nm) in a similar GSSG configuration [77] (portrayed in Figure 5d). These coupons were suspended using a resist tethering technique, obviating the need for etched pillar trenches. Also here, yield was demonstrated by showing a sample with hundreds of suspended coupons and 26 printed coupons, from which only one was faulty due to mechanical breakage. The extended interaction length enabled a significantly lower Vπ of 3.2 V, whereas the ER remained comparable to previous designs at 31 dB. However, the absence of tapers at the SiPho–LN interface resulted in a higher insertion loss of 4.7 dB. High‐speed characterisation (plotted in Figure 5d) revealed a 3 dB EOE bandwidth exceeding 35 GHz. As is common in the literature, this bandwidth figure excludes the initial roll‐off below 5 GHz, which is likely attributable to impedance mismatch between the electrodes and probes or the use of a low‐resistivity substrate [108].

With 2.4 mm long and 100 μm wide coupons, Badri et al. demonstrated that wide LN coupons (2.4 mm × 100 μm × 300 nm) could also be suspended using the resist tether to the substrate technique [76]. A single‐ended GSG configuration was adopted, offering compactness at the potential expense of bandwidth. An electrode gap of 9 μm was chosen to mitigate absorption losses, resulting in an IL of 8 dB without tapers. The ER was 27 dB, and the Vπ was 43.6 V (Figure 5c), comparable to the initial single‐arm demonstrator. No high‐speed measurements were reported.

A further demonstration focused on the scalability of the μTP process, employing both loss‐measurement structures and MZMs as demonstrators [74]. Unlike prior lab‐scale SiN platforms, this work utilised a SiPho chip from a multiproject wafer (MPW), enabling a multilayer stack with both Si and SiN for passive routing. This architecture reduced transition losses without requiring LN tapers, which are challenging to fabricate. The total IL was 4.7 dB, of which only 0.8 dB was attributed to the heterogeneous SiN/LN interface. A total of 217 LN coupons were printed across a full commercial wafer with 99% yield; only visually contaminated sites remained unprinted. In the MZM section, 7 mm × 30 μm × 300 nm coupons were printed in a GSSG configuration on several O‐band modulators, with an effective interaction length of 6.6 mm. All 33 MZMs exhibited ERs around 30 dB and Vπ values near 4 V (with a 5 μm electrode gap). One MZM, printed on a high‐resistivity substrate, was characterised at high speed and, as can be seen in the lower plot of Figure 5e, exhibited a 3 dB EOE bandwidth exceeding 70 GHz, without initial roll‐off. In view of higher throughput, also an initial demonstration was given of array‐printed coupons, printing 28 (4 × 7) 1 mm × 30 μm coupons in a single printing movement, the result of which is shown at the top of Figure 5e. In this case, picking and printing alignment is done in a similar way compared to single coupon printing. Since the PDMS stamp is mounted on a glass plate, local vertical compliance is provided, whereas lateral stiffness is conserved, keeping the relative alignment between all coupons constant. Since the transfer operation maintains the pitch between the coupons, aligning one coupon aligns all coupons from the array to be printed. Mainly, a parameter to consider for transfer of multiple coupons in a single operation is the agreement between the pitch between the stamp posts and the pitch between the coupons. For large arrays (> 1 ${\text{cm}}^{2}$), a prior calibration of the fabricated stamp pitch compared to the stamp design is realised, aiming for the compensation of the variation from design to fabrication. Transfer of larger areas in a single step is a work in progress.

The demonstration by Su et al. utilised MZMs within a 2× 2 switch architecture rather than as stand‐alone devices [92]. Four C‐band MZMs were fabricated using 2 mm long LN coupons in a GSG configuration. The Vπ was measured at 20.9 V (with a 7 μm electrode gap), as depicted in Figure 5f, and the average IL of the complete switch circuit was 4.7 dB. The ER of the devices is estimated to be more than 30 dB, deduced from the insertion loss and cross‐talk measurements of the 1 × 2 switch.

The most recent demonstration by Murai et al. demonstrate the feasibility of patterned devices for LN μTP on SiPho. 3 mm long coupons were transferred to a Si circuitry, where both LN waveguides are patterned in the transferred device that form the arms of an MZM in GSG configuration. With an ER of 23 dB and an IL of 1 dB in C‐band operation, the device exhibits a Vπ of 14 V. High‐speed operation shows an EOE bandwidth of more than 67 GHz, limited by the measurement equipment.

All metrics are gathered in Table 3, sorted by date of appearance.

**TABLE 3:** Overview of all transfer printed heterogeneous LN on SiPho Mach–Zehnder modulators.

Ref	Demo	WL [nm]	Length [mm]	Electrode gap [μm]	IL [dB]	ER [dB]	Vπ [V]	EOE BW [GHz]
[71]	MZM	1550	0.9	6.5	7^a^	12	61.1	N.M.
[99]	MZM	1550	2	6	3.3^a^	39	14.8	55
[77]	MZM	1550	10	6	4.6	31	3.2	35
[76]	MZM	1550	2.4	9.2	8	27	43.6	N.M.
[74]	MZM	1310	6.6	5	4.7^a^	30	4.0	> 70
[92]	MZM switch	1550	2	7	4.7^a^	N.M.	20.9	N.M.
[102]	MZM	1550	3	6	1	23	14	> 67

^a^ SiPho routing included.

#### 4.1.2 Ring Modulators

In addition to travelling‐wave modulators, ring modulators have also been manufactured for exploiting the electro‐optical properties of LN. Owing to their resonant nature, ring modulators enable more compact and energy‐efficient devices, making them particularly attractive for applications where low footprint and reduced driving voltage are prioritised over ultrahigh‐speed performance. These resonant structures have been employed not only for modulation but also for the generation of broadband electro‐optic frequency combs [109] and as tunable optical filters [110, 111].

Two different approaches for μTP of TFLN onto a SiPho platform for ring resonators have been mentioned in the literature: both patterned and unpatterned TFLN coupons can be used for the fabrication of a heterogeneous LN/SiPho ring resonator. An initial demonstration of a heterogeneous TFLN‐on‐SiN ring resonator through μTP was achieved by using a patterned LN slab, in which the ring is defined through shallow etching of the LN, as shown in Figure 6a [72]. This patterned slab, measuring 100 μm × 100 μm, is transfer printed onto a SiN bus waveguide, where precise alignment allows for coupling from the SiN waveguide to the LN ring. By using this patterned waveguide structure in the printed LN slab, the mode overlap in the LN increases compared to an unpatterned slab, and it allows for smaller bend radii. The loaded Q‐factor of the resulting device, measured at C‐band, was 39,000. As can be seen in Figure 6a, this device was not metallised and thus not used for electro‐optic modulation. To fully exploit the high Pockels coefficient of LN, the resonator geometry is ideally racetrack‐shaped rather than circular as electro‐optic modulation is most efficient along the crystallographic *Z*‐axis. The racetrack configuration, when used as a modulator, will be discussed in more detail later on.

**FIGURE 6:**
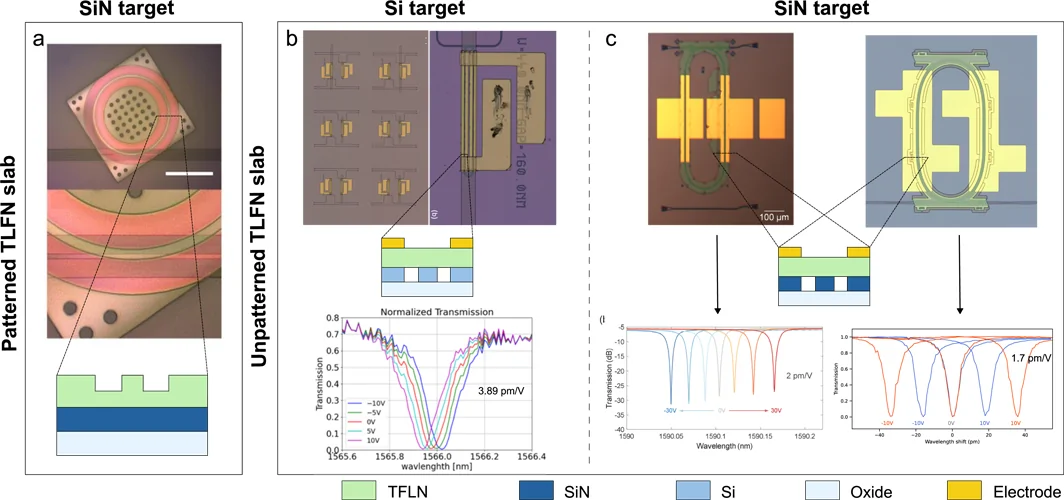
Heterogeneous ring modulators. (a) Optical microscope picture of a patterned LN ring resonator transfer printed on a SiN bus waveguide, with cross‐section [72]. (b) Optical microscope picture of the micro‐transfer printed LN‐on‐Si ring modulator, with cross section and optical transmission for different modulation voltages [104]. (c) Optical microscope pictures from the microtransfer printed LN‐on‐SiN ring modulators [100] (left) and [90] (right), with cross‐section and corresponding transmission spectra for different modulation voltages. Panel (a) is adapted with permission from ref. [72] Optica Publishing Group under licence CC BY 4.0; Panel (b) is adapted with permission from ref. [104] and the right column in panel (c) is adapted with permission from ref. [100] American Chemical Society, whereas the right column in panel (c) is adapted with permission from ref. [90] Optica Publishing Group.

Another, more popular approach for the fabrication of TFLN‐on‐SiPho ring resonators through heterogeneous integration is transfer printing of an unpatterned LN slab onto a Si/SiN resonator circuit [90, 100, 104]. In this configuration, the mode is guided by the underlying SiPho platform but is also partially confined in both the LN slab on top, allowing for a heterogeneous mode. Furthermore, there is no need for low‐loss etching of the LN or strict alignment during the μTP process as in the previously discussed approach.

In 2024, Tan et al. demonstrated the first heterogeneously integrated TFLN‐on‐Si ring modulator through μTP as shown in Figure 6b [104]. The high index contrast of silicon waveguides enabled a compact racetrack modulator, onto which a 50 μm × 700 μm TFLN slab was transfer‐printed. After μTP, metallisation was applied to complete the device. The resulting modulator exhibits an extinction ratio between 20 and 37 dB, a Q‐factor of 11,180 and a tuning efficiency of 3.89 pm/V for modulation of both arms.

A downside of using a Si platform for electro‐optical modulation is the presence of two‐photon absorption (TPA), which results in a degrading performance of the TFLN‐on‐Si modulator. SiN on the other hand, exhibits negligible TPA process compared to Si, making it increasingly attractive for heterogeneous integration. However, the lower refractive index of SiN necessitates larger bending radii, which complicates the fabrication of compact ring modulators using the same approach as for silicon. The larger bend radii, namely, require wider TFLN slabs to be printed onto the SiN circuit, posing challenges for coupon suspension. Although narrow long TFLN coupons can typically be suspended using support structures along their sides, wider coupons often require more complex methods, such as the pillar approach, which involves etching holes into the membrane [72, 73].

Recently, two similar papers demonstrated an alternative approach to overcome the need for wide suspended coupons by adopting the shape of the coupon to the SiN circuitry [90, 100]. This results in racetrack‐shaped TFLN coupons, which can be suspended through the same methods as narrow linear geometries. Su et al. demonstrated the fabrication of microring coupons with dimensions over 230 μm x 2000 μm using this approach [100]. Both Su et al. and De Jaeger et al. transfer‐printed TFLN coupons with sizes of 250 μm × 750 μm [100] and 250 μm × 500 μm [90], respectively, onto SiN racetrack resonators for the fabrication of TFLN‐on‐SiN modulators. Figure 6c shows the resulting devices as well as their tuning efficiencies. The modulators achieved tuning efficiencies of 3.5 pm/V (double‐arm modulation) and 2 pm/V (single‐arm modulation), extinction ratios above 20 dB and loaded Q‐factors of 285,000 and 150,000, for [90, 100], respectively. Although both studies report similar device performance, the coupon fabrication strategies differ. Su et al. manufacture the coupons by using LN tethers to the side, which simplifies the coupon fabrication process, but causes the need for a custom PDMS stamp, in order to pick only the coupon and not the side structures. In contrast, De Jaeger et al. encapsulate the coupon with resist and adds pillar support before release. This approach allowed to the LN within the racetrack to be removed during etching, enabling transfer printing with a standard rectangular stamp, in this case 250 μm × 500 μm‐sized rather than a custom‐shaped one. Nonetheless, these different approaches indicate the versatility of the μTP process, where different methods of coupon fabrication yield similar results. An overview of all mentioned rings is gathered in Table 4.

**TABLE 4:** Overview of all transfer printed heterogeneous LN on SiPho ring modulators.

Ref	Electrode gap [μm]	Loaded Q‐factor	Tuning efficiency [pm/V]	Coupon size [μ$m^{2}$]	EO bandwidth [GHz]	SiPho platform
[72]	NA	32,000	NA	100 × 100	NA	Si
[103]	NA	77,000	NA	100 × 60	NA	SiN
[104]	3–4	11,180	3.89^a^	500 × 50	16	Si
[100]	6	150,000	2	250 × 750	NR	SiN
[90]	8	285,000	3.5^a^	250 × 500	1.18	SiN

^a^ Double‐arm modulation.

#### 4.1.3 Fabry–Pérot Modulators

In this section on μTP‐integrated LN‐on‐SiN modulators, the Fabry–Pérot (FP) modulator is discussed as the final device type. To date, FP modulators have been reported in only two studies [76, 101], although similar implementations have been demonstrated on other platforms [112, 113]. These resonance‐based devices are anticipated to offer a performance trade‐off between Mach–Zehnder modulators (MZMs) and ring modulators, in terms of bandwidth and footprint. In the work by Badri et al., two FP modulator variants were presented: a conventional design and a side‐coupled configuration. Both were fabricated using the same μTP coupons as those employed for the MZM demonstrators, sized 2.4 mm × 100 μm × 300 nm. However, rather than relying on interferometric splitting and recombination in the underlying SiN, resonant cavities are created using distributed Bragg reflectors (DBRs). C‐band operation was demonstrated for both variants, with insertion losses below 7 dB and extinction ratios of 8.5 and 15 dB were reported for the standard and side‐coupled devices, respectively. With metal electrode gaps of 15 and 15.2 μm, a Vπ of 65.2 and 130.2 V was measured. However, it should be noted that no high‐speed characterisation was performed.

In the work of Ananthachar et al., a conventional design was fabricated. Coupons sized 1.2 mm × 70 μm × 200 nm were integrated on a SiN platform where DBRs form a resonant cavity. When operating in C‐band, only a Q‐factor was reported to be 52,200.

### 4.2 Nonlinear Devices

Silicon photonics inherently lacks a $\chi^{\left(2\right)}$ nonlinearity due to the inversion symmetry of both Si and SiN, which prohibits efficient frequency conversion via three‐wave mixing. Although symmetry‐breaking techniques can induce an effective second‐order response [114, 115], the resulting conversion efficiencies remain limited. In contrast, TFLN intrinsically supports a strong $\chi^{\left(2\right)}$ nonlinearity and its wide transparency range, low‐loss propagation and poling capability make it an attractive nonlinear platform. Single‐pass conversion efficiencies have reached up to 4600%/Wcm^2^ at C‐band [116] and 33,000%/Wcm^2^ at 900 nm [117]. By incorporating the PPLN into a resonator, this can be further enhanced to 5,000,000%/W [118].

Through μTP, the efficient three‐wave mixing processes can be added to the existing SiPho components whilst preserving scalability and CMOS‐compatibility. This unlocks a range of new functionalities on the platform, from advanced light sources, such as optical parametric oscillators [49] and efficient optical squeezers [119], to practical applications including self‐referencing of SiN‐based Kerr frequency combs [120].

To enable efficient frequency conversion, the phase‐matching condition must be satisfied. Two approaches are commonly adopted for single‐pass waveguides: quasi‐phase‐matching (QPM) and modal phase‐matching (MPM). In the former, the lithium niobate is periodically poled to create a $\chi^{\left(2\right)}$ grating, where the poling period is chosen to compensate the index difference between the lowest order modes at the involved wavelengths (Figure 7b). In the latter, a less‐confined higher order mode is targeted at the smaller wavelength that has an identical index to the lowest order spatial mode at the fundamental wavelength (Figure 7g).

**FIGURE 7:**
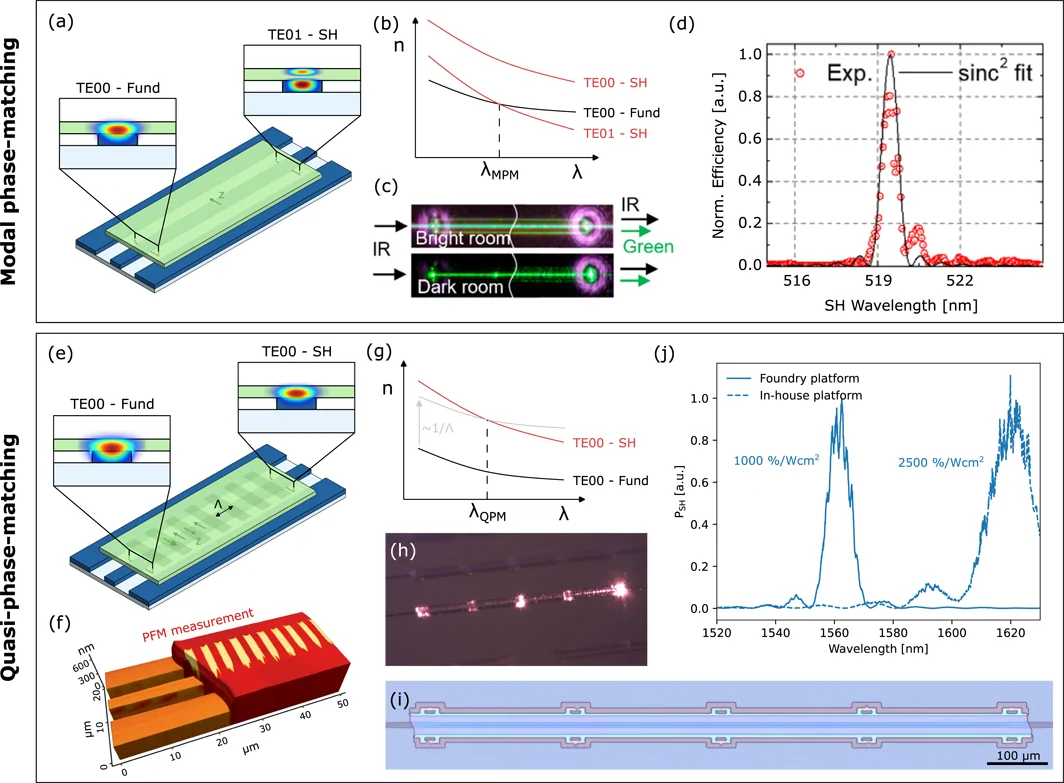
Heterogeneous frequency converters. (a) Sketch of the nonlinear device for modal phase‐matching. (b) Dispersion relations of the involved modes for modal phase‐matching. (c) Photographs of generated green light in LN/SiN waveguides. (d) Measured SHG frequency conversion. (e) Sketch of the nonlinear device for quasi‐phase‐matching. (f) Combined AFM and PFM measurement of a PPLN/SiN waveguide. (g) Dispersion relations of the involved modes for quasi‐phase‐matching. (h) Photograph of generated 780 nm light inside the nonlinear waveguide. (i) Optical microscope image of a PPLN/SiN device. (j) Measured SHG frequency conversion. Panels (c–d) are reproduced with permission from ref. [105] Optica Publishing Group; Panels (f, h–j) are reproduced with permission from ref. [75] under licence CC BY 4.0.

Both MPM and QPM have recently been realised in TFLN slabs that are microtransfer printed onto SiN waveguides to create hybrid waveguides. Sumon et al. have shown green light generation by frequency doubling of 1040 nm (Figure 7c,d) [105]. They rely on phase‐matching between the TE00 mode at the fundamental and the TE01 mode at the second harmonic. The operating wavelength is controlled through the waveguide dimensions, for example, by sweeping the SiN width. A normalised conversion efficiency of 42.5%/Wcm^2^ is obtained with a bandwidth of 0.8 nm for a 2.4 mm interaction length. Although the conversion efficiency is lower than that of LNOI rib waveguides [48], attributed to propagation losses of the TE01 mode, their approach circumvents the demanding fabrication required for periodically poling TFLN. However, the trade‐off is that the second harmonic is generated in a higher‐order spatial mode, which is inherently more difficult to handle.

The alternative strategy of QPM has been demonstrated by Vandekerckhove et al. [75]. In this approach, TFLN is periodically poled prior to μTP, thereby avoiding the need for high voltages on the SiPho platform. A poling period of 3.5 μm enables phase‐matching between the TE00 modes at 1550 and 775 nm. Periodic poling introduces an additional degree of freedom, allowing the waveguide dimensions to be reserved for higher‐order dispersion engineering, albeit at the expense of more complex fabrication. QPM second‐harmonic generation was demonstrated on two SiN platforms, an in‐house and a foundry platform, achieving conversion efficiencies of 2500%/Wcm^2^ and 1000%/Wcm^2^, with corresponding bandwidths of 15 and 8 nm, respectively, for 1 mm interaction lengths (Figure 7f–j).

Furthermore, the superior process control of SiPho is leveraged to address the extreme sensitivity of quadratic nonlinear waveguides to fabrication variations [121, 122]. Reproducibility is demonstrated on several chips of a 200 mm silicon photonics wafer and combined with a wafer‐level tuning mechanism, leads to a strategy for scalable integrated quadratic nonlinearities. Lastly, the platform is used to demonstrate spontaneous parametric down‐conversion, establishing printable entangled photon‐pair sources.

### 4.3 New Directions

#### 4.3.1 LN Integration for Optomechanics

So far, the focus has been put on electro‐optic interactions such as high‐speed modulation and frequency conversion. However, lithium niobate also exhibits strong piezoelectricity, making it a widely used platform for acousto‐optic devices, including modulators [123], MEMs resonators [124] and beam‐steering systems [125]. Through μTP, LN's piezoelectric effect can be locally integrated with other platforms that feature high‐quality phonon environments, thereby enhancing acousto‐optic functionality.

Si is a widely used optomechanical platform, valued for its low acoustic losses and strong optomechanical coupling [126]. When combined with the electro‐mechanical coupling of transfer‐printed LN, it enables efficient interaction between electronic and optical channels mediated by phonons, all within a compact footprint. This hybrid approach is particularly attractive for applications, such as quantum transducers, where the quantum state of an RF photon can be coherently converted into an optical photon, establishing optical interconnects between superconducting quantum systems [127]. Jiang et al. have demonstrated such a system that generates entanglement between optical and microwave photons [128]. By microtransfer printing a patterned LN slab, they avoid etching of the Si surface, which would significantly reduce the optical quality factors. A similar electro‐optical approach is taken by Burger et al. to design a bidirectional release‐free quantum transducer [129].

Another platform that leverages acousto‐optic interactions is diamond. It has demonstrated high optomechanical Q‐factors [130], and the presence of superior colour centres positions it as a leading candidate for solid‐state quantum memories, where the spins of the colour centres can be efficiently driven via coherent surface acoustic waves (SAWs) [131, 132]. Since diamond itself is not piezoelectric and cannot generate SAWs, transfer‐printed LN is used to provide this functionality. For instance, Xu et al. demonstrate coherent driving of an electronic spin on a LN–on–diamond platform [133]. Following a similar approach, Malik et al. report an LN phononic delay line transfer‐printed onto diamond as a step towards phonon‐mediated hybrid quantum systems [134].

#### 4.3.2 Micro‐Transfer Printing of Lithium Tantalate

Lithium tantalate (LT) has recently been introduced in the literature as an alternative candidate for heterogeneous integration of electro‐optic properties onto SiPho platforms [135, 136, 137]. LT is believed to offer distinct advantages, including lower dielectric loss, higher thermal stability and reduced photorefractive effects. Despite these merits, LT has historically received less attention in integrated photonics due to its slightly lower electro‐optic efficiency and the lack of commercially available thin‐film LT substrates. Only recently have advances in thin‐film preparation enabled LT to be explored as a viable material for heterogeneous integration.

A first demonstration by Niels et al. showcased the successful printing of LT coupons onto SiPho modulators, thereby extending the material versatility of the μTP platform [135]. In this demonstration, eight 7 mm × 30 μm × 300 nm coupons were printed, using BCB as a buffer layer, on four O‐band MZMs in GSSG configuration on the same MPW platform as used by Niels et al. [74]. The resulting devices exhibited an IL of 6.7 dB, with contributions attributed to the modulating arms propagation and transition loss of 2.9 dB and the rest to the Si routing around. An ER exceeding 28 dB was achieved, and with a metal gap of 5.5 μm, a Vπ of 3.5 V was reported. EOE bandwidth measurements indicated performance beyond 70 GHz.

In another study, published in the same year, LT coupons of 4 mm × 70 μm × 300 nm were printed onto C‐band GSG MZMs [136]. In this work, 34 coupons were transferred, and statistical analysis revealed an average IL of 0.82 dB and an ER of 30 dB. The same metal gap of 5.5 μm was employed, yielding a Vπ of 7.6 V. 3 dB EOE bandwidths exceeding 67 GHz were reported as illustrated in Figure 8b.

**FIGURE 8:**
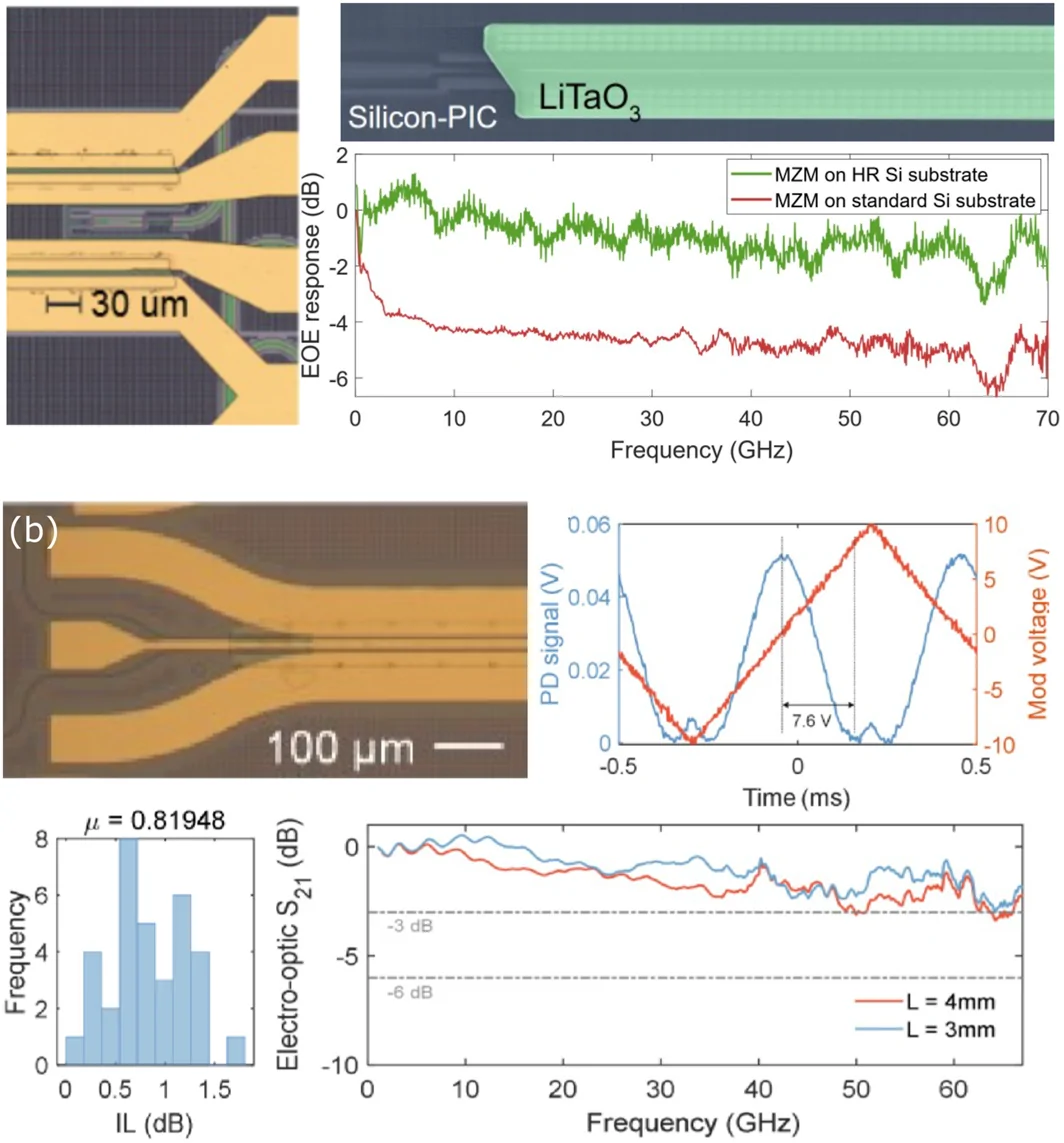
Lithium tantalate heterogeneous integration through microtransfer printing. (a) Microtransfer printed GSSG MZM and its high‐speed behaviour for low‐ and high‐resistivity (HR) substrate [135] and (b) microtransfer printed GSG MZM and its characterisation metrics [136]. Panel (a) is adapted with permission from ref. [135] under licence CC BY 4.0 and panel (b) is adapted with permission from ref. [136].

Both demonstrations of heterogeneous integration of LT on SiPho and their specs are listed in Table 5.

**TABLE 5:** Overview of all printed LT coupons for heterogeneous LT/SiPho platforms.

Ref	Platform	Coupon size (L × W × H)	Adhesive	# Coupons	Demo	WL [nm]	Length [mm]	Electrode gap [μm]	IL [dB]	ER [dB]	Vπ [V]	EOE BW [GHz]
[135]	LT/SiN	7 mm × 30 μm × 300 nm	BCB	8	MZM	1310	6.6	5.5	6.7^a^	28	3.5	> 70
[136]	LT/SiN	4 mm × 70 μm × 300 nm	None	34	MZM	1550	4	5.5	0.82	30	7.6	> 67

^a^ SiPho routing included.

## 5 Scalability and Volume Manufacturing

Integrated photonics‐based solutions have demonstrated significant potential in recent years across various fields, including data communications, telecommunications and quantum computing, by leveraging their compact form factor, low energy consumption and cost‐effectiveness. However, realising their transition into practical applications requires the large‐scale deployment of integrated photonic devices, encompassing both low‐volume manufacturing (LVM) for prototyping and high‐volume manufacturing (HVM) for industrial uptake. In this context, CMOS‐compatible platforms based on Si and SiN remain the most viable, owing to their process maturity and scalability. Nevertheless, intrinsic limitations in modulation speed and nonlinear conversion efficiency constrain their competitiveness for advanced functionalities. As previously discussed, several demonstrations of LN integration on SiPho platforms via μTP have been reported, offering a pathway to overcome these limitations. Furthermore, in this context, the integration needs to progress towards 200 and 300‐mm wafer‐scale fabrication to make this heterogeneous integration approach compatible with research and development (R&D) as well as industrial applications. A simplified schematic of the heterogeneous platform production flow is illustrated in Figure 9.

**FIGURE 9:**
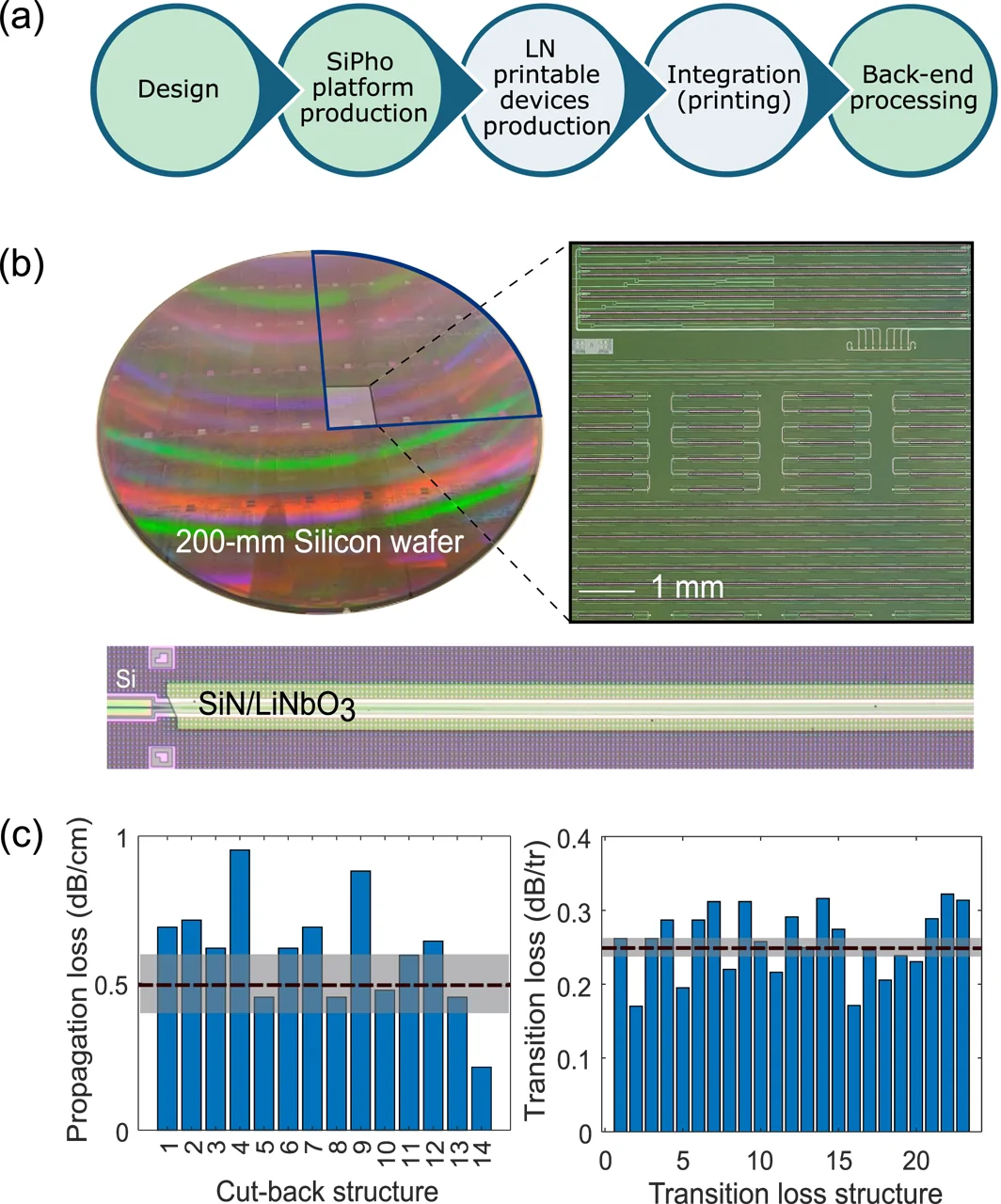
(a) Schematic flow for the high‐volume manufacturing of a heterogeneous SiPho‐LN platform. (b) Demonstration of LN printing on a 200‐mm SiPho wafer [74]. (c) Passive measurements of a 200‐mm LN‐SiPho platform [74]. Panels (b) and (c) are adapted with permission from ref. [74] Optica Publishing Group.

The following section provides a module‐by‐module overview of the current status within this flow (also gathered in Table 6), alongside an assessment of the implications for HVM compatibility.

**TABLE 6:** Module status and compatibility with HVM.

Module	Status	Compatibility HVM
Design	Available	Yes
SiPho platform	Available	Yes
LN devices	Demonstrated	Yes
Integration	Demonstrated	Yes
Back‐end	Available	Yes

5.1

**Design:** This module adheres to standard CMOS photonic design principles. The inclusion of active heterogeneous components is fully compatible with existing process design kits (PDKs), requiring only the addition of alignment markers for μTP. The module is HVM‐compatible.

5.2

**SiPho platform production:** This module follows conventional SiPho fabrication protocols. For advanced integration schemes (e.g., 3D architectures), recesses in the cladding are required to accommodate heterogeneous elements. The module is HVM‐compatible.

5.3

**LN printable devices production** **:** This module is specific to the μTP approach. Although coupon‐based fabrication has been demonstrated, the establishment of a robust supply chain for printable LN devices remains ongoing. Nonetheless, several LNOI wafer‐scale foundries, such as CCRAFT, Lightium, Hyperlight and Liobate, have shown high‐yield processing capabilities, rendering this module promising for future HVM.

5.4

**Integration (printing):** This module is specific to the μTP integration approach. Wafer‐scale demonstrations of III–V μTP integration have been reported [138] and also preliminary results for LN [74]. To mitigate lithium contamination risks, the process must be conducted in dedicated facilities [139]. Although the concept is technically feasible for HVM, the supply chain infrastructure remains under development.

5.5

**Back‐end processing:** After the printing, wafers may be encapsulated in dielectric cladding, with subsequent back‐end processing performed in standard fabrication lines. This module is fully compatible with HVM.

The production and scalability of LN integration via microtransfer printing depend on two main factors. The first is technical, involving the printing process itself. Recent advances have demonstrated high potential, with 99% printing yield on full 200‐mm wafers and alignment deviations within 500 nm (3σ) [74]. A printing loop time of only 1 min and successful printing of coupon arrays further confirm the technique's feasibility. The second factor is strategic, relating to logistics and industry engagement. Although many supporting modules are mature, the production and high‐volume manufacturing of printable LN devices are not yet established. An open question remains regarding the future manufacturing paradigm for large‐scale TP‐based integration. One possible model is the emergence of ‘super foundries’ that include separate dedicated process lines for CMOS materials and thin‐film electro‐optics materials as well as integration capabilities, all within a single fabrication facility. However, this approach would require a very dense concentration of advanced manufacturing infrastructures and complexity. An alternative model involves specialised transfer printing foundries, where wafers fabricated at different foundries worldwide are subsequently transferred to a centralised site for transfer printing integration and back‐end processing. This latter approach introduces several potential challenges, including wafer shipping and handling, contamination risks during wafer‐out and wafer‐in processes and ensuring compatibility and continuity in downstream back‐end processing. Nevertheless, the need for silicon photonics platforms that go beyond the current state of the art has been increasingly driven by industry in recent years, and several R&D‐oriented demonstrations have already been realised. Their advancement now hinges on the interest and investment strategies of R&D and industrial stakeholders.

## 6 Future Outlook and Conclusion

Microtransfer printing has rapidly evolved from a niche research technique into a promising candidate for industrial‐scale heterogeneous integration of LN onto SiPho platforms. Its unique ability to selectively place high‐performance LN devices onto CMOS‐compatible substrates with high alignment precision and minimal thermal budget opens up new avenues for scalable photonic integration. Although challenges remain, such as establishing a robust supply chain for printable LN devices and further reducing insertion losses, the progress made in recent years is remarkable. Demonstrations of wafer‐scale μTP with yields exceeding 99%, alignment deviations within 500 nm (3σ) and successful printing of hundreds of coupons highlight the technique's maturity and readiness for high‐volume manufacturing. Looking ahead, μTP is poised to play a central role in the next generation of photonic integrated circuits. Its flexibility allows for the cointegration of diverse material platforms, enabling hybrid systems that combine the low‐loss routing of SiN, the dense integration of Si, and the high‐speed modulation and nonlinear capabilities of LN. This opens the door to advanced applications in telecommunications, quantum photonics, frequency conversion and optomechanics, for which already several demonstrators have been reported. As the ecosystem around μTP continues to grow, with increasing involvement from foundries, tool vendors and research institutions, the technique is expected to transition from R&D labs to mainstream photonic manufacturing. With continued innovation and investment, μTP could become a foundational technology for heterogeneous photonics, unlocking unprecedented performance and functionality in compact scalable formats.

## Author Contributions

**Margot Niels:** conceptualisation, data curation, writing – original draft. **Tom Vandekerckhove:** conceptualisation, data curation, writing – original draft. **Lisa De Jaeger:** conceptualisation, data curation, writing – original draft. **Maximilien Billet:** conceptualisation, writing – original draft, data curation, supervision. **Bart Kuyken:** conceptualisation, writing – original draft, supervision, resources, funding acquisition.

## Funding

We want to thank the European Space Agency for funding under the E/0365‐70 ‐ NAVISP, LEO Project and the FWO and F.R.S.‐FNRS under the Excellence of Science (EOS) program (40007560).

## Data Availability

The data that support the findings of this study are available from the corresponding author upon request. The data are not publicly available due to privacy or ethical restrictions.

## Endnotes

^1^
The term ‘EOE bandwidth’ refers to the electro‐optic–electrical bandwidth, typically measured using a vector network analyser and photodiode as illustrated in a schematic in Figure 5d. A 3 dB EOE bandwidth corresponds to a 6 dB drop in the optical modulation response, which means that the 3 dB EOE bandwidth is equivalent to the 6 dB EO bandwidth. The EO‐bandwidth is often obtained by using an OSA [106, 107].
